# Comparison of native and non‐native predator consumption rates and prey avoidance behavior in North America and Europe

**DOI:** 10.1002/ece3.6932

**Published:** 2020-10-27

**Authors:** Ayse Gül Ünlü, John J. Obrycki, Roman Bucher

**Affiliations:** ^1^ Conservation Ecology Philipps‐Universität Marburg Marburg Germany; ^2^ Department of Entomology College of Agriculture, Food and Environment University of Kentucky Lexington Kentucky USA

**Keywords:** coccinellidae, co‐evolution, cue avoidance, invasive species, predator–prey interactions

## Abstract

Novel predator–prey interactions can contribute to the invasion success of non‐native predators. For example, native prey can fail to recognize and avoid non‐native predators due to a lack of co‐evolutionary history and cue dissimilarity with native predators. This might result in a competitive advantage for non‐native predators. Numerous lady beetle species were globally redistributed as biological control agents against aphids, resulting in novel predator–prey interactions. Here, we investigated the strength of avoidance behavior of the pea aphid (*Acyrthosiphon pisum*) toward chemical cues of native lady beetles and non‐native Asian *Harmonia axyridis* and European *Coccinella septempunctata* and *Hippodamia variegata* in North America, hypothesizing that cues of non‐native lady beetles induce weaker avoidance behavior than cues of co‐evolved native lady beetles. Additionally, we compared aphid consumption of lady beetles, examining potential predation advantages of non‐native lady beetles. Finally, we compared cue avoidance behavior between North American and European pea aphid populations and aphid consumption of native and non‐native lady beetles in North America and Europe. In North America, pea aphids avoided chemical cues of all ladybeetle species tested, regardless of their origin. In contrast to pea aphids in North America, European pea aphids did not avoid cues of the non‐native *H. axyridis*. The non‐native *H. axyridis* and *C. septempunctata* were among the largest and most voracious lady beetle species tested, on both continents. Consequently, in North America non‐native lady beetle species might have a competitive advantage on shared food resources due to their relatively large body size, compared to several native American lady beetle species. In Europe, however, non‐native *H. axyridis* might benefit from missing aphid cue avoidance as well as a large body size. The co‐evolutionary time gap between the European and North American invasion of *H. axyridis* likely explains the intercontinental differences in cue avoidance behavior and might indicate evolution in aphids toward non‐native predators.

## Graphical abstract

Here, we compared the role of cue avoidance behavior of pea aphids and lady beetle body size for the invasion success of lady beetles in North America and Europe. In contrast to North American pea aphids, European pea aphids did not avoid cues of the non‐native *Harmonia axyridis*; in addition, consumption rates were strongly correlated with body size of lady beetles. Thus, we suggest that predation advantages of non‐native predators due to missing antipredator behaviors of prey might diminish with time, whereas body size‐related competition advantages over smaller native and non‐native predators could sustainably benefit large, non‐native predators.

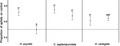

## 1 INTRODUCTION

Predator–prey interactions shape ecosystems via density‐ and trait‐mediated effects (Murdoch et al., 2003; Preisser et al., 2005). Density‐mediated effects result in the elimination of prey individuals by a predator leading to reduced prey population densities (Murdoch et al., 2003). Prey adapt to the selection pressure of predators by evolving traits that increase the survival during predator attacks (i.e., antipredator behaviors). However, changes in these plastic traits can come at a fitness cost (i.e., non‐consumptive effects; Lima & Dill, 1990; Peacor & Werner, 2000). Fitness costs of trait‐mediated effects can reduce prey population densities to a similar extent as density mediated effects (Preisser et al., 2005). Both, density‐ and trait‐mediated effects can expand into adjacent trophic levels (i.e., trophic cascades or trait‐mediated indirect interactions; Ohgushi et al., 2012; Terborgh & Estes, 2010).

Predator‐induced changes in prey behavior (i.e., antipredator behavior) can enhance prey survival upon predator attacks, interfering with the detection, encounter, and/or capture of prey (Lima, 1998). Prey species have sensory mechanisms to detect and recognize cues of co‐evolved predators, to effectively respond to a predator attack (Lima & Dill, 1990; Rosier & Langkilde, 2011). Predator cues serve as sensory information for prey, to recognize co‐evolved predators and induce antipredator behaviors. Cues that are involved in interspecific communication can be visual, vibrational cues, and olfactory cues (Fischer et al., 2001; Hermann & Thaler, 2014). Chemical cues left by predators persist for some time in nature and can be an indicator of predator presence and predation risk (Bucher et al., 2014). Missing co‐evolution of predator and prey can lead to a lack of detection and recognition mechanisms of predator cues by prey (Cox & Lima, 2006). Non‐native predators can therefore benefit from a novelty advantage due to lacking or inappropriate antipredator response by prey, leading to higher predation pressure (Sih et al., 2010). Non‐native predators can consequently have stronger consumptive effects and weaker non‐consumptive effects on prey populations, compared to co‐evolved predators. If cues of non‐native and native predator species are similar then a similar response can be expected by prey, regardless of predator origin (Sih et al., 2010). Cue similarities between predator species can therefore lower the impact on prey densities compared to dissimilar non‐native predators (Sih et al., 2010). In addition, cues of closely genetically‐related species tend to be more similar compared to cues of distantly genetically related species, for example, chemical cues within lady beetle genera are more similar than between genera (Magro et al., 2010).

Lady beetles leave species‐specific chemical cues on the plant tissue, which are persistent and long‐lasting (Dixon and Dixon, 2000). The species‐specific chemical cues left in the tracks of lady beetles consist of cuticular hydrocarbons (Kosaki & Yamaoka, 1996) that serve for water proofing (Menzel et al., 2019) and mediate intra‐ and interspecific communication (Doumbia et al., 1998; Hemptinne et al., 1998; Menzel et al., 2019; Ninkovic et al., 2013). Recent studies revealed that the presence of lady beetle chemical cues on host plants can induce avoidance behavior in herbivores; for example, the Asian citrus psyllid *Diaphorina citri* (Hemiptera: Psyllidae) (Seo et al., 2018), the bird cherry‐oat aphid *Rhopalosiphum padi* (Ninkovic et al., 2013), as well as the pea aphid *Acyrthosiphon pisum* (both Hemiptera: Aphididae) (Ünlü et al., 2020).

The pea aphid *Acyrthosiphon pisum* Harris (Hemiptera: Aphididae) consists of numerous distinct biotypes, being adopted to host plants in its local range (Peccoud et al., 2009a; Peccoud, et al., 2009b). Originally of Palearctic origin, North American populations of pea aphids co‐evolved in agricultural fields with native predators for over a century (Thomas, 1878).

Lady beetles (Coleoptera: Coccinellidae) have a history of being globally introduced as biological control agents for decades (Harmon et al., 2007). Lady beetles are predators of several pest species (e.g., aphids and coccids), thus providing a valuable ecosystem service in agriculture (Caltagirone & Doutt, 1989; Obrycki & Kring, 1998). Among the introduced lady beetle species, the European species *Coccinella septempunctata* and *Hippodamia variegata* have been released for biological control of aphids in North America (Angalet et al., 1979; Ellis et al., 1999). The earliest establishment of *C. septempunctata* in North America dates back to 1973 (Angalet & Jacques, 1975). The establishment history of *H. variegata* began in 1984 in North America (Gordon, 1987). Similarly, the Asian *Harmonia axyridis* was introduced as a biological control agent in North America and Europe (Tedders & Schaefer, 1994; Trouve et al., 1997). The introduction of the Asian *H. axyridis* in North America started in 1916 (Gordon, 1985), but its earliest establishment was in 1988 (Chapin & Brou, 1991).

In Europe, *H. axyridis* was introduced in 1995 and the establishment period started in 2000–2001 (Brown et al., 2011). *Coccinella septempunctata* and the Asian *H. axyridis* are relatively large and highly voracious compared to common native aphidophagous species (Elliott et al., 1996; Hoki et al., 2014; Ünlü et al., 2020). Moreover, both species interfere with native trophic interactions associated with a lady beetle species decline in the non‐native range, due to resource competition and intraguild predation (Alyokhin & Sewell, 2004; Ware et al., 2009), absence of natural enemies (Roy et al., 2011), high abundance (Horn, 1991; Koch, 2003) and high fecundity (Kajita & Evans, 2010) and are therefore classified as invasive species (Roy & Brown, 2015). The contribution of cue avoidance behavior of aphids confronted with non‐native and native chemical lady beetle cues to the invasion success of non‐native lady beetles remains to be examined.

In this study, we deployed a multi‐species approach to compare differences in cue avoidance behavior of a North American population of pea aphid (*Acyrthosiphon pisum*) confronted with chemical cues of the Asian lady beetle species *Harmonia axyridis*, the European lady beetle species *Coccinella septempunctata* and *Hippodamia variegata* and three North American lady beetle species, *Coleomegilla maculata*, *Coccinella novemnotata* and *Hippodamia convergens*. Our species set consisted of two native and non‐native species of the same genus Coccinella and Hippodamia, referred to as congeneric, expecting similarities between chemical cues (Magro et al., 2010). In addition, we compared aphid consumption rates between all lady beetles tested. We hypothesized (a) missing avoidance behavior of *A. pisum* confronted with cues of the non‐native *H. axyridis*, intermediate avoidance behavior confronted with cues of congeneric non‐native species (*Coccinella septempunctata* and *Hippodamia variegata*), due to potential cue similarities and strongest avoidance behavior toward native lady beetle cues. (b) We expected higher aphid consumption of the larger non‐native lady beetle species *H. axyridis* and *C. septempunctata* compared to smaller lady beetle species, regardless of origin. In addition, cue avoidance and consumption experiments were conducted in Europe, using a European pea aphid population as prey and non‐native *H. axyridis*, native *Coccinella septempunctata* and *Hippodamia variegata* as predators. We subsequently compared cue avoidance behavior and consumption of North American and European pea aphids confronted with lady beetle species occurring on both continents. We expected (c) missing avoidance behavior toward *H. axyridis* cues in North America and Europe. Moreover, we expected the avoidance behavior of European pea aphids toward cues of *C. septempunctata* and *H. variegata* to be stronger in the native European range compared to avoidance behavior of North American aphids. (d) We expected no differences in aphid consumption of *H. axyridis*, *C. septempunctata*, and *H. variegata* between North America and Europe, due to body size‐related food demands.

## 2 MATERIAL AND METHODS

### 2.1 Study species North America

The North American pea aphid *Acyrthosiphon pisum* (Harris) (Hemiptera: Aphididae) colony consisted of individuals maintained in a colony, which started in 1985 at Iowa State University, Ames, Iowa, USA and individuals collected in Lexington, Kentucky in 2003. The colony was maintained in the laboratory (at Iowa State University and the University of Kentucky) on broad bean plants (*Vicia faba*, variety Windsor). They were kept in cages with six to eight pots containing five plants each. Plants were replaced weekly to guarantee a fresh food supply for aphids. Aphids were maintained in the laboratory in climate chambers (22°C ± 1 and a photoperiod of light 16 hr: dark 8 hr) and in a climatized laboratory (22°C ± 1 and a photoperiod of light 16 hr: dark 8 hr). The lady beetle species *Coccinella septempunctata and Colleomegilla maculata* were collected in April 2018 in alfalfa fields and in field margins at an agricultural research field station of the University of Kentucky in Lexington, Kentucky, USA. The overwintering generation of *Hippodamia convergens* was obtained from Rincon Vitova Insectaries, Ventura, CA, USA, in April 2018 and stored at low temperatures (5°C). Female and male beetles of these species were subsequently paired in 0.24‐liter paper cartons, provided with water and fed ad libitum with pea aphids, and frozen *Ephestia kuehniella* (Zeller) (Lepidoptera: Pyralidae) eggs (Beneficial Insectary, Redding, CA, USA). Egg clusters laid by individual females were collected and placed into a Petri dish. When larvae hatched, they were separated into glass vials, sealed with cotton, provided with water and fed ad libitum with pea aphids and frozen *E. kuehniella* eggs until pupation. Individuals of *Harmonia axyridis* were field collected in the pupal stage in May‐June and kept in Petri dishes (circumference: 9.4 cm × height: 1.6 cm) until the adult beetles emerged. *Hippodamia variegata* individuals were collected from an alfalfa field in Le Roy, IL, USA in May/June. In June, *C. novemnotata* was purchased in the larval stage (Lost Ladybug Project, Cornell University, Ithaca, New York 14,850), since no individuals were found in Kentucky and separately kept in glass vials (see above) until they developed to adults. The adult lady beetles were subsequently sorted by species and stored in plastic boxes. They were provided with water and fed ad libitum with pea aphids, *A. pisum* and frozen *E. kuehniella* eggs and kept at 22 ± 1°C, at a photoperiod of light 16 hr: dark 8 hr. Voucher specimens were preserved in Ethanol (70%) and stored under −7 ± 1°C at the Department of Entomology (Animal Pathology Building), at the University of Kentucky.

### 2.2 Study species Europe

The European pea aphid colony was obtained from the Julius‐Kühn Institut in Braunschweig, Germany, which had been maintained in the laboratory since at least 2007. The aphids were reared on broad bean *V. faba* (v. Sutton Dwarf, Kings Seeds, Manchester) in plastic containers (10.0 × 13.5 × 6.5 cm) covered with gaze for aeration in climate chambers (20 ± 1°C, L:D 16:8 and 65% relative humidity). Aphids were supplied with fresh plants, weekly. The lady beetle species *H. axyridis and C. septempunctata* were collected in June‐September 2017 and *H. variegata* in 2018 in grasslands around Marburg, Germany. Ladybeetles were kept in small groups, separated by species, in Petri dishes (circumference: 9.4 cm × height: 1.6 cm), fed ad libitum with *A. pisum* and were kept at (20 ± 1°C, L:D 16:8 and 65% relative humidity). The data for the European predation and cue avoidance experiments used for the intercontinental comparison are a subset of the European comparison published by Bertleff et al. (2020).

### 2.3 Cue avoidance experiments

Beetles were sexed prior to the experiments, to ensure a gender‐balanced design (ten male and ten female beetles). Lady beetles were sexed by the morphological differences on the terminal sternites of females and males (Costopoulos et al., 2014; Harmon et al., 2008; Hurst et al., 1999; Nichols & Neel, 1974; Riddick & Schaefer, 2005; Stellwag & Losey, 2014). Double leaflets of *Vicia faba* were cut in two halves, one control and one treatment leaflet, and separately placed into round Petri dishes (circumference: 3.5 cm × height: 1.0 cm). A single lady beetle adult was placed on the treatment leaflet in the Petri dish for cue deposition (e.g., footprints, feces) and subsequently removed after 12 hr. The control leaflet remained without a lady beetle. The control and the treatment leaflet were randomly assigned and placed into the center of each half of a round Petri dish (circumference: 9.4 cm × height: 1.6 cm); the treatment leaflet was placed into one half of the Petri dish, while the control leaflet was placed into the opposite half. Ten adult aphids were released into the center of each Petri dish, which were capped to prevent aphids from leaving the experimental arena. The duration of the preparation of the experimental set‐up (between removal of the lady beetle from the treatment leaf and the release of aphids) was 60 min. The number of aphids on the control and treatment leaflet was counted after 0.25, 0.5, 1.0, 1.5, 2.0, and 3.0 hr. Twenty replicates were conducted per species in the laboratory under 25.10 ± 0.20°C and artificial lightning. The leaf choice experiments in Europe were identical except that lady beetle individuals were not sexed prior to the experiments, but randomly chosen.

### 2.4 Predation experiments

Lady beetles were sexed by morphological differences prior to the experiments, accounting for potential intraspecific predation differences of female and male beetles. A single lady beetle adult was subsequently placed into a small round Petri dish (circumference: 3.5 cm × height: 1.0 cm) and starved for 24 hr prior to the experiment. Thirty pea aphids (second to third nymph stage) were counted and placed with a brush into a round Petri dish (circumference: 9.4 cm × height: 1.6 cm). A single lady beetle was randomly assigned to a Petri dish containing aphids, which was capped. Aphid predation was quantified by counting the remaining aphids in the Petri dish after 6 hr. In North America, we freeze‐killed (−7 ± 1°C) lady beetle individuals after the experiments and measured body width (widest horizontal distance of closed elytra) and body length (elytral apex to pronotal apex) of all beetles used for the predation experiments under a stereomicroscope. We followed the procedure of Obrycki et al. (1998) to obtain elliptical body area for individual beetles (body area (mm^2^) = (*π* × 0.5 × body length (mm) × 0.5 × body width (mm)). Overall, 20 replicates (ten females, ten males) were conducted per species, in the laboratory under 25.41 ± 0.19°C and artificial lightning. The predation experiments in Europe were identical, except that lady beetle individuals were randomly chosen and not sexed prior to the experiments.

### 2.5 Statistical analysis

For the cue avoidance experiments in North America and the intercontinental comparison, aphid counts on each leaflet were analyzed as proportions (aphids on control leaf versus treatment leaf). We only considered aphids that made a distinct choice of control or the treatment leaflet. We applied a GLMM with a binomial error distribution to analyze differences between cue donator species identity (i.e., different lady beetle species) on aphid leaf choice. We included cue donator species identity as fixed effects and experimental unit (Petri dish identity) and an observation level random effect (OLRE) as random effects (to account for repeated measurements and overdispersion). We obtained statistical parameters for the fixed effects via ANOVA (*χ*2‐test) from the R‐package car (Fox & Weisberg, 2019). Pairwise differences between cue donator species identity were analyzed with a Tukey's contrast test for comparison of means with a Holm correction, to account for familywise error rates, using the glht‐function from the multcomp R‐package (Hothorn et al., 2008). We subsequently tested for equal distribution of aphids on control versus. treatment leaf (i.e., if aphids avoid lady beetle cues of the respective cue donator), by applying a GLMM with binomial error distribution. Our fixed effects included species identity and experimental unit (i.e., repeated measurements) and ORLE (accounting for overdispersion) as random effects.

Differences of predation rates in North America after 6 hr and body area were respectively analyzed with a Games–Howell post hoc test, following a Welch's ANOVA (*F* test) accounting for heteroscedasticity. To test the effects of lady beetle species identity, gender and body area on predation rates, we conducted a GLM with lady beetle species identity, gender and body area as fixed effects with a quasi‐poisson error distribution. Statistical parameters for the fixed effects were obtained via ANOVA (*χ*2‐test). The intercontinental predation differences between lady beetle species were analyzed with a Games–Howell post hoc test, following a Welch's ANOVA (*F* test). All statistical analyses were performed with the statistical software R, Version 3.4.0 (R Development Core Team, 2017).

## 3 RESULTS

### 3.1 Cue avoidance in North America

**Figure 1:**
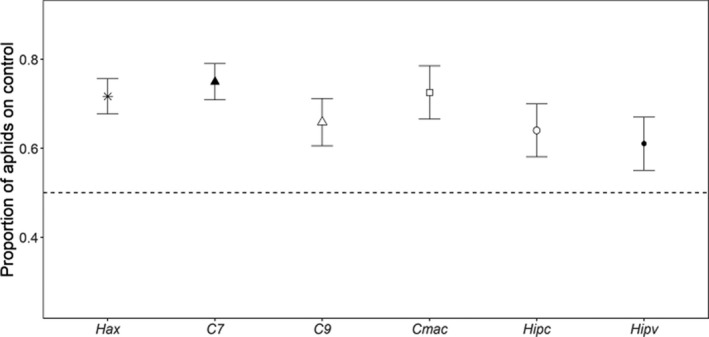
Proportion of pea aphids on cue‐free control leaflets (mean ± *SE*) in North America. Pea aphids avoided leaflets with chemical cues of the native lady beetle species (unfilled symbols) *Coccinella novemnotata* (*C9*), *Coleomegilla maculata* (*C. mac*), *Hippodamia convergens* (*Hipc*) and the non‐native lady beetle species (filled symbols) *Harmonia axyridis* (*Hax*), *Coccinella septempunctata* (*C7*) and *Hippodamia variegata* (*Hipv*) (*p* ≤ .02, respectively)

Aphid leaf choice did not differ between cues of the different lady beetle species (GLMM; *χ* ^2^ = 5.80, *df* = 5, *p* = .33). Aphids avoided leaves previously occupied by all species (Figure 1; *H. axyridis*: *z*_95_ = 4.03, *p* < .01; *C. septempunctata*: *z*_95_ = 4.89, *p* < .01; *C. novemnotata*: *z*_95_ = 3.13, *p* < .01; *Col. maculata*: *z*_95_ = 4.63, *p* < .01; *H. convergens*: *z*_95_ = 2.72, *p* < .01 and *H. variegata*: *z*_95_ = 2.35, *p* = .02).

### 3.2 Aphid consumption by lady beetles in North America

**Figure 2:**
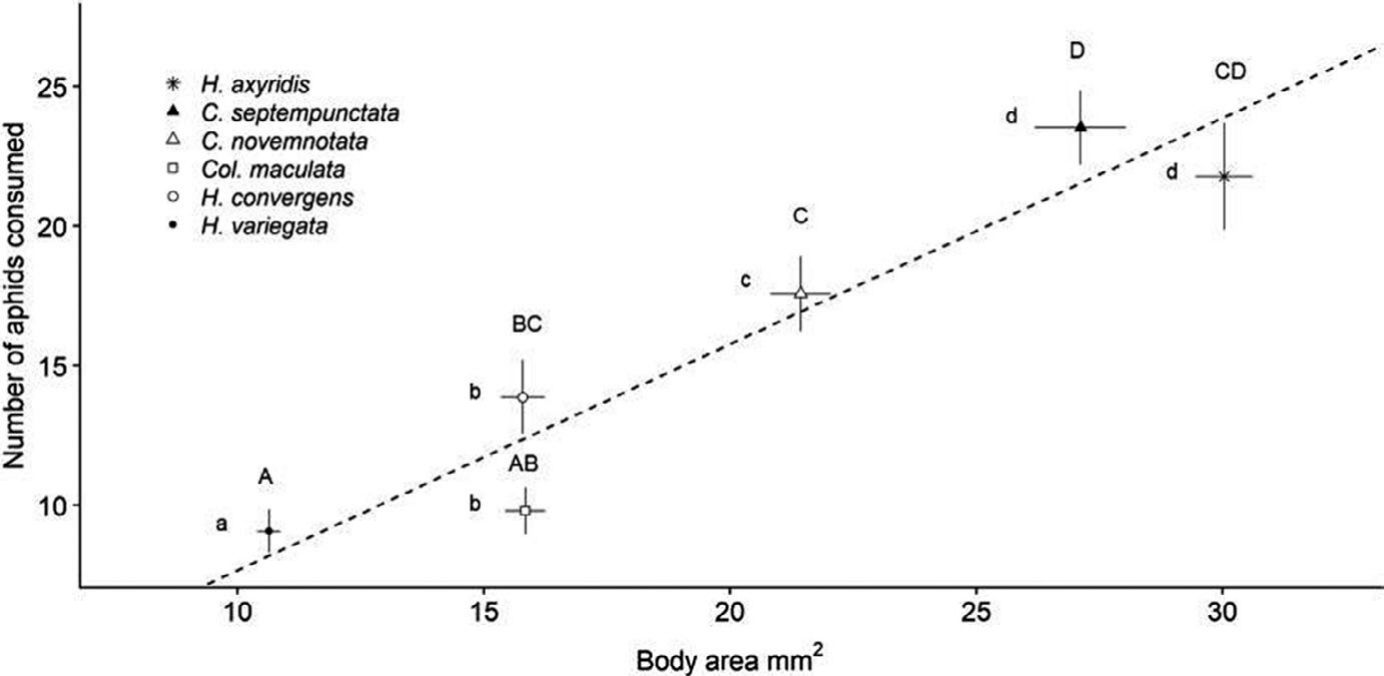
Number of pea aphids consumed after 6 hr (mean ± *SE*) and body area (mm^2^; mean ± *SE*) of native (unfilled symbols) and non‐native lady beetle species (filled symbols): Different uppercase letters indicate statistical differences of aphid consumption and different lowercase letters indicate statistical differences in body area between species based on a Games–Howell post hoc test (*p* < .05, same letters do not differ significantly). Dotted regression line (*y* = 0.8133*x* − 0.4305; *R* ^2^ = 0.54) shows the linear relationship between aphid consumption and body area

The number of aphids consumed after 6 hr differed among lady beetle species (Welch's ANOVA; *F* = 26.13, *df* = 5, *p* < .01). Aphid consumption by *C. septempunctata* and *H. axyridis* did not significantly differ (Games–Howell post hoc test (GH); *p* = .97; Figure 2). There were no differences in aphid consumption between *C. novemnotata* and *H. axyridis* (GH, *p* = .48); Aphid consumption of *C. novemnotata* was lower compared to *C. septempunctata* (GH, *p* = .03). Aphid consumption of *C. septempunctata* and *H. axyridis* was respectively higher compared to *H. convergens*, *Col. maculata*, and *H. variegata* (GH; *p* ≤ .02).

Body area differed between lady beetle species (Welch's ANOVA; *F* = 248.34, *df* = 5, *p* < .01). There were no body area differences between *C. septempunctata* and *H. axyridis* (*p* = .11; Figure 2) The remaining species (*C. novemnotata*, *H. convergens*, *Col. maculata*, and *H. variegata)* were respectively smaller than *C. septempunctata* and *H. axyridis* (GH; *p* < .01).

*Hippodamia convergens*, *Col. maculata*, and *H. variegata* were smaller than *C. novemnotata*, (GH; *p* < .01). There were no size differences between *H. convergens* and *Col. maculata* (GH; *p* = 1.00). *Hippodamia variegata* was smaller than *Col. maculata* and *H. convergens* (GH; *p* < .01). Consumption rates of lady beetles can be explained by species identity (GLM; *χ* ^2^ = 23.18, *df* = 5, *p* < .01), beetle gender (GLM; *χ* ^2^ = 26.32, *df* = 5, *p* < .01), and beetle body size (GLM; *χ* ^2^ = 6.23, *df* = 5, *p* = .01).

### 3.3 Intercontinental comparison of aphid cue avoidance

**Figure 3:**
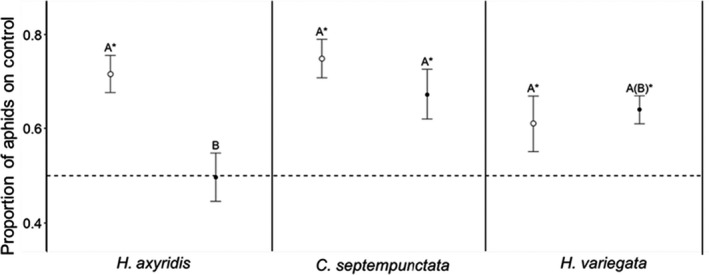
Proportion of aphids on cue‐free control leaflets (mean ± *SE*) in North America (unfilled symbols) and Europe (filled symbols). Asterisk indicates significant avoidance behavior of leaflets with chemical cues of lady beetle species (*p* < .05). Different letters indicate statistical differences between species based on Tukey's contrast test (*p* < .05)

Avoidance behavior of local pea aphids differed between cues of different lady beetle species (GLMM; *χ* ^2^ = 30.56, *df* = 5, *p* = .01). Avoidance behavior of European aphids was weaker when confronted with *H. axyridis* cues compared to avoidance behavior of North American aphids, *C. septempunctata* cues from both continents, and North American *H. variegata* cues (Tukey's contrasts test (TCT); *p* < .01, respectively; Figure 3). Avoidance behavior was marginaly higher when confronted with European *H. variegata* cues compared to European *H. axyridis* cues (TCT; *p* = .08). No differences of avoidance behavior were observed between the remaining species (TCT; *p* > .29, respectively).

In Europe, *A. pisum* showed no avoidance confronted with cues of *H. axyridis* (*z*_95_ = −1.12, *p* = .26; Figure 3), but avoidance of *C. septempunctata* cues (*z*_95_ = 5.31, *p* < .01) and *H. variegata* cues (*z*_95_ = 2.59, *p* < .01). In North America *A. pisum* showed avoidance to *H. axyridis* cues (*z_95_* = 4.429, *p* < .01), *C. septempunctata* cues (*z*_95_ = 3.85, *p* < .01), and *H. variegata* cues (*z*_95_ = 2.44, *p* < .01).

### 3.4 Intercontinental comparison of lady beetle consumption rates

**Figure 4:**
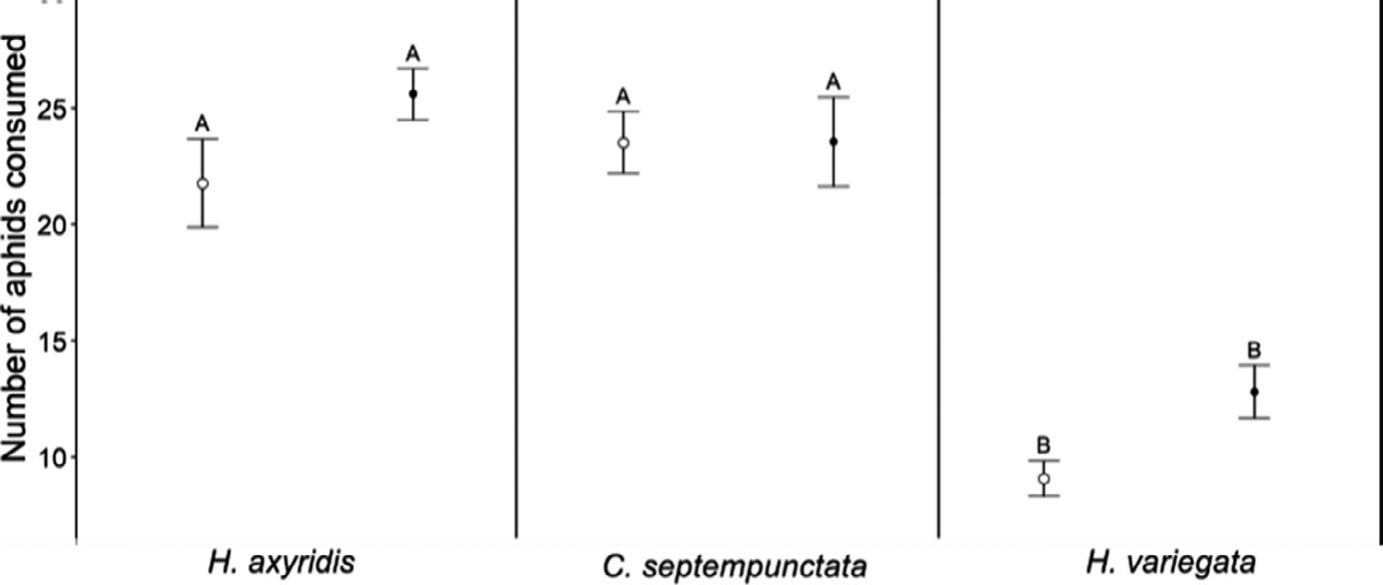
Number of pea aphids consumed after 6 hr (mean ± *SE*) of lady beetle species in North America (unfilled symbols) and Europe (filled symbols): Different letters indicate statistical differences of aphid consumption of different lady beetle species based on a Games–Howell post hoc test (*p* < .05, same letters do not differ significantly)

Predation rates differed among lady beetle species (Welch's ANOVA; *F* = 42.38, *df* = 5, *p* < .01). North American *H. variegata* consumed significantly fewer aphids than North American and European *C. septempunctata* (GH; *p* < .01, respectively) and North American and European *H. axyridis* (GH; *p* < .01, respectively). Moreover, *European H. variegata* showed lower consumption rates compared to North American and European *C. septempunctata* (GH; *p* < .01, respectively) and North American and European *H. axyridis* (GH; *p* < .01, respectively). European and North American aphids were consumed to a similar extent by *H. axyridis* from North America or Europe (GH; *p* = .52). Moreover, consumption rates did not differ between European *C. septempunctata* and North American *C. septempunctata* (GH; *p* = 1.00) as well as between European *H. variegata* and North American *H. variegata* (GH; *p* = .10). *Coccinella septempunctata* and *H. axyridis* consumption rates did not differ significantly in North America (GH; *p* = .97) and in Europe (GH; *p* = .94; Figure 4).

## 4 DISCUSSION

North American pea aphids avoided chemical cues of all lady beetle species tested, regardless of lady beetle origin. In contrast to North American pea aphids, European pea aphids did not avoid cues of the non‐native *H. axyridis*. Consumption rates were strongly correlated with body size of lady beetles. On both continents, *C. septempunctata* and *H. axyridis* were the largest species tested and consumed the most aphids.

Pea aphids in Europe and North America avoided chemical cues of native lady beetles. These findings are in line with previous studies on predator–prey interactions showing that chemical cues of predators induce avoidance behavior in herbivores (Ninkovic et al., 2013; Seo et al., 2018; Ünlü et al., 2020). Chemical cues of predators can persist in the environment and indicate potential predation risk of a nearby predator (Kats & Dill, 1998). By avoiding sides with chemical cues of predators, prey can increase its survival (Lima & Dill, 1990). According to the “landscape of fear,” prey shifts to sites with low predation pressure by avoiding sites with high predation risk (Laundré et al., 2001). A previous study on the avoidance behavior of the Asian citrus psyllid, *Diaphorina citri*, toward lady beetle trail chemicals demonstrated that chemical cues of lady beetles can not only interfere with the feeding activity of prey but additionally reduce oviposition (Seo et al., 2018). Another study confirms that prey can discriminate between risky and suitable feeding sides by demonstrating that the Colorado potato beetle, *Leptinotarsa decemlineata* reduces feeding on potato leaves covered with predator cues, compared to a cue‐free control (Hermann & Thaler, 2014). Moreover, prior studies showed that disturbances in pea aphid behavior induced by predator cues can lead to increased searching behavior for suitable feeding sides and consequently decreased feeding times, resulting in reproductive costs (Nelson, 2007; Nelson et al., 2004). However, predator cues, covering the surrounding area, can lead to aphid dispersal to sites with less predator pressure and consequently reduce survival costs through immediate predator consumption (Roitberg et al., 1979). Chemical cues deposited by lady beetles were trail chemicals and feces. According to Ninkovic et al. (2013), a similar avoidance response of aphids was observed, when feces were excluded from the experiments and only the trail chemicals of lady beetles were considered, suggesting that the effect of feces on the avoidance behavior of aphids is negligible (Ninkovic et al., 2013). Thus, our findings suggest that chemical lady beetle cues can equally repel aphids and might subsequently serve as a signal for predation risk. Future field studies should target the effects of lady beetle cues on aphid dispersal and the reproduction of aphids, to shed light on the non‐consumptive effects on aphid populations.

The avoidance of non‐native *H. axyridis* cues by North American pea aphids might be explained by the strong selection pressure by non‐native predators on native prey, leading to the evolution of antipredator behaviors (Carthey & Blumstein, 2018). In a maritime system, for example, the intertidal snail *Littorina obtusata* responded to the increasing predation pressure of the intertidal crab (*Carcinus maenas*), which was expanding its range, with rapid morphological change of shell forms (Seeley, 1986). Moreover, research on cue avoidance behavior in mammals showed that native common ringtail possum (*Pseudocheirus peregrinus*) recognized and subsequently avoided olfactory cues of the invasive European red fox (*Vulpes vulpes*), within a few generations of co‐evolution (Anson & Dickman, 2013).In addition, our results show that *H. axyridis* is among the largest and most voracious predators tested; since large predators can pose a greater thread for prey, this can result in stronger antipredator responses by prey (see Binz et al., 2014).

The cue avoidance experiments in North America and Europe revealed differences in avoidance behavior toward cues of the non‐native *H. axyridis*. In contrast to North America, the European pea aphids showed no avoidance behavior toward *H. axyridis* cues, indicating predator–prey naïveté (sensu Cox & Lima, 2006, Sih et al., 2010). North American and European pea aphid populations used for the experiments were maintained for more than 10 years in the laboratory and were expected to have experienced only low densities of established non‐native lady beetle species in the field. However, pea aphids and non‐native lady beetles coexisted prior to the establishment of non‐native lady beetle species in North America (Gordon, 1985; Harmon et al., 2007). According to regional studies, *H. axyridis* was first reported in 1992 in Kentucky (Cottrell & Yeargan, 1998). In contrast, pea aphids have a longer history in Kentucky, for example, a study on the parasitation rate of the non‐native parasitoid *Aphidius smithi* on pea aphids was conducted from 1967 to January 1970 confirming the pest status of pea aphids in agricultural environments (Pass & Parr, 1971). Although the regional co‐occurrence between *H. axyridis* and *A.pisum* might not exceed 10 years, the migration history of non‐native *H. axyridis* as well as pea aphids throughout North America must be considered (Brown et al., 2011; Lamb & MacKay, 1979), suggesting prior co‐evolutionary history in agricultural sides beyond Kentucky. Specifically, in North America, *H. axyridis* was released in multiple agricultural landscapes starting in 1916 (Gordon, 1985) and was repeatedly reintroduced as a biological control agent to control agricultural pest species (Gordon, 1985; Lombaert et al., 2014; Tedders & Schaefer, 1994). Thus, the frequency of predator–prey encounters and the length of co‐evolutionary time since introduction can be decisive for the evolution of antipredator behaviors (Gérard et al., 2014; Nelson, 2007). In North America, we suggest that North American pea aphid populations might have evolved cue avoidance behavior toward *H. axyridis* during the longer co‐evolutionary time spend in shared agricultural fields (Gordon, 1985). In contrast, co‐evolutionary history of *A. pisum* is shorter with *H. axyridis* populations in Europe, compared to that of North American populations (Brown et al., 2011; Gordon, 1985). In Europe, *H. axyridis* was introduced in the 1990s and the establishment period ranged from 2000–2007 (Brown et al., 2011; Klausnitzer, 2002). The European aphid laboratory colony was established, when spread and establishment of *H. axyridis* started in Central Europe (Brown et al., 2011). Consequently, the European aphid colony has experienced low H. axyridis densities, if any. Our results thus provide a snapshot of initial interactions between pea aphids and *H. axyridis* in Europe. To enhance our comprehension of evolutionary changes in non‐native predator–prey interactions, long‐term studies are required, starting with the initial introduction of the non‐native predator (Anton et al., 2020; Mallon et al., 2015).

The repelling substances within the chemical cues of lady beetles, inducing cue avoidance behavior in pea aphids, are unknown and remain to be identified. It might be possible that rather than evolutionary changes, cue similarities between chemical cues of *H. axyridis* and native lady beetles might have resulted in avoidance behavior of *A. pisum* when *H. axyridis* was introduced to North America. However, in Europe, chemical cues of the non‐native *H. axyridis* were not avoided, indicating dissimilarity to cues of native lady beetle species. Thus, similarly to chemical cues of *H. axyridis* in Europe, chemical cues of *H. axyridis* might differ from cues of native lady beetle species in North America.

In North America, pea aphids avoided chemical cues of non‐native *C. septempunctata* and *H. variegata* to a similar extend as cues of the congeneric native *C. novemnotata* and *H. convergens*. Chemical cue similarities between congeneric non‐native *C. septempunctata* and *H. variegata* and native *C. novemnotata* and *H. convergens* in North America might contribute to the equally strong avoidance behavior in pea aphids. Here, cues of non‐native and native lady beetle species of the same genera can be similar (Magro et al., 2010) and consequently a similar response can be expected in prey, regardless of predator origin (Sih et al., 2010). However, the degree of cue similarity between the tested lady beetle species remains open and needs further attention. In addition, according to the “multipredator hypothesis,” prey retains evolved antipredator behaviors toward extinct predators in the presence of remaining predators (Blumstein, 2006). Pea aphids in Europe avoided chemical cues of the co‐evolved *C. septempunctata* and *H. variegata* to a similar extent, as pea aphids in North America. Thus, in North America chemical cue avoidance toward non‐native European *C. septempunctata* and *H. variegata* might be a retained innate antipredator response of pea aphids, which evolved prior to the introduction of pea aphids to North America in the beginning of the 19th century and remained due to the presence of congeneric native predators with potentially similar cues (e.g., *C. novemnotata* and *H. convergens*). Overall, the “multipredator hypothesis” in combination with chemical cue similarities of congeneric species might explain cue avoidance of congeneric non‐native *C. septempunctata* and *H. variegata* and native *C. novemnotata and H. convergens* in North America, as well as the similar strength of avoidance behavior between European and American pea aphids. Still further studies are required to evaluate retained antipredator behaviors in non‐native insect species beyond their native range. Next to *H. axyridis*, *C. septempunctata* was the largest and most voracious non‐native lady beetle species in North America tested. The regional co‐occurrence of pea aphids and *C. septempunctata* used in the experiments might exceed 10 years, since, for example, Buchele et al. (1992) reported *C. septempunctata* appearing in research plots in Kentucky in 1987. Similar to *H. axyridis,* the introduction and establishment of *C. septempunctata* in North America dates decades back (Angalet & Jacques, 1975; Kajita et al., 2012). This alternatively suggests that avoidance behavior of pea aphids toward chemical cues of *C. septempunctata* might have newly evolved in North America, due to the increased predation pressure by this large and voracious predator (see Binz et al., 2014).

In North America, the non‐native *C. septempunctata* and *H. axyridis* were the largest lady beetles tested and consumed the most aphids, compared to smaller lady beetle species. Food consumption increases with body mass, due to increasing metabolic requirements (Brose et al., 2008), confirming the positive relationship between lady beetle body area and aphid consumption rates. The successful establishment of a large predator can depend on, that is, predator size and prey availability (Crookes et al., 2019). In North America, *C. septempunctata* was primarily considered as a non‐native biological control agent on pest species, due to large size and voraciousness (Elliott et al., 1996). Predation advantages of invasive *H. axyridis* over the smaller native lady beetle species *Cycloneda sanguinea* on shared pest species were attributed to a dominance in intraguild interactions, wider dietary range, higher voracity, and larger size (Michaud, 2002). Moreover, we found that the smaller sized, non‐native *H. variegata* consumes a lower number of aphids, compared to *C*. septempunctata and *H. axyirids* in North America. Thus, asymmetric competition advantages over smaller native and non‐native species can benefit the larger non‐native *H. axyridis* on both continents and non‐native *C. septempunctata* in North America (Hoki et al., 2014; Michaud, 2002). Furthermore, a recent study found that the efficiency of resource utilization was comparatively higher in invasive *H. axyridis* than in native *H. convergens*, when allometric scaling was considered. In addition, aphid handling time was lower and maximum consumption rate was higher in *H. axyridis*, compared to native *H. convergens*, indicating that the invasive *H. axyridis* is the dominating competitor (Crookes et al., 2019). Additionally, *C. septempunctata* and *H. axyridis* are both successful intraguild predators of native coccinellids in their invaded range (Pell et al., 2008; Snyder et al., 2004), which is not only beneficial in dietary terms, but also reduces competition on shared resources (Yasuda et al., 2004, Dixon and Dixon, 2000). Overall, body size and correlated physiological and/or behavioral traits of invasive species can significantly contribute to a competition advantage toward native and non‐native predators (Hemptinne et al., 2012; Kajita & Evans, 2010; Michaud, 2002; Obrycki et al., 1998).

Based on the current status of *H. axyridis* and European lady beetle species in North America, the two larger species *H. axyridis* and *C. septempunctata* have spread all over the United States within few decades, compared to the smaller *H. variegata*, *Propylea quatuordecimpuncata*, and *Adalia bipunctata*, which kept a more local distribution in the North East (and North West for *A. bipunctata*; Gordon, 1985, Lost Ladybug Project 2020: www.lostladybug.org). Among other characteristics, we argue that a large body size can contribute to the invasion success of lady beetles due to increased food demands and thus stronger competition. Analyzes of a 24‐year dataset in southwestern Michigan revealed only significant declines in the relatively small *Col. maculata* and *A. bipunctata* (Bahlai et al., 2015). Likewise, *A. bipunctata* showed stronger declines in the presence of *H. axyridis* compared to other native lady beetle species, in Europe (Roy et al., 2012). In contrast, *C. novemnotata* maintained an ecological foothold in the face of invasion by the equally sized *C. septempunctata* (Evans, 2017). Thus, we suggest that lady beetle body size might be a good predictor for their invasion potential in areas beyond their native ranges.

In contrast to body size, differences in predator avoidance can diminish with time. Here, North American aphids but not European aphids avoided cues of the Asian *H. axyridis*. Such evolutionary adaptations can contribute to so called “boom‐bust dynamics”: invaders go through an initial outbreak before declining to a lower population size (Simberloff & Gibbons, 2004; Strayer et al., 2017). So far, evidence for a decline in *H. axyridis* populations is restricted to microsatellite effective population estimates (Sethuraman et al., 2018). In the long term, the adaptations of antipredator behaviors by aphids toward non‐native predators might result in a stable co‐existence within the native community and may consequently lead to a more harmless situation relative to the current impact of *H. axyridis*. Evolutionary responses to non‐native predators have important consequences for ecological studies aiming to elucidate the underlying mechanism of biological invasion such as a lack of avoidance behavior: Although lacking avoidance behavior toward non‐native predators during early stages of biological invasions benefits the non‐native predator, the mechanisms might no longer be detectable at later stages due to co‐evolution. Our results in concert with lady beetle distribution data in North America and in Europe indicate that relative lady beetle body size is a key predictor of the invasion success of non‐native lady beetle species, but also for native lady beetle species that are at particular risk if they co‐occur with non‐native lady beetles.

## 5 CONCLUSION

Missing avoidance behavior of European pea aphids toward chemical cues of non‐native *H. axyridis* suggests that non‐native predators can benefit from chemical cue novelty resulting in a lack of antipredator behavior of prey, during early stages of biological invasions. In contrast, North American pea aphids showed avoidance behavior toward *H. axyridis* cues, suggesting adaptations of avoidance behavior against voracious, non‐native predators. Overall, predation advantages of non‐native predators due to missing antipredator behaviors of prey might diminish with time, whereas body size‐related competition advantages over smaller native and non‐native predators could sustainably benefit large, non‐native predators.

## CONFLICT OF INTEREST

None declared.

## AUTHOR CONTRIBUTIONS

**Ayse Gül Ünlü:** Formal analysis (lead); investigation (lead); methodology (supporting); visualization (lead); writing–original draft (lead); writing–review and editing (equal). **John Obrycki:** Project administration (supporting); resources (supporting); supervision (supporting); writing–review and editing (supporting). **Roman Bucher:** Conceptualization (lead); funding acquisition (lead); methodology (lead); project administration (lead); supervision (lead); writing–review and editing (equal).

## Data Availability

We archived our data on the Dryad data repository: https://doi.org/10.5061/dryad.47d7wm3bt.
